# Potential Gradient‐Driven Dual‐Functional Electrochromic and Electrochemical Device Based on a Shared Electrode Design

**DOI:** 10.1002/advs.202401948

**Published:** 2024-05-20

**Authors:** Gang Xu, Wei Zhang, Guangjun Zhu, Huan Xia, Hanning Zhang, Qian Xie, Peng Jin, Haoyu Zhang, Chengjie Yi, Ruqian Zhang, Lingfeng Ji, Tao Shui, Nosipho Moloto, Wei She, ZhengMing Sun

**Affiliations:** ^1^ Jiangsu Key Laboratory of Advanced Metallic Materials School of Materials Science and Engineering Southeast University Nanjing 211189 China; ^2^ State Key Laboratory of High Performance Civil Engineering Materials Southeast University Nanjing 211189 China; ^3^ Department of Civil and Mechanical Engineering Technical University of Denmark Kgs Lyngby 2800 Denmark; ^4^ Molecular Science Institute School of Chemistry University of the Witwatersrand Private Bag 3, Wits 2050 Johannesburg 2000 South Africa

**Keywords:** electrochromic device, gradient potential, self‐powered, shared electrode, zinc‐ion battery

## Abstract

The integration of electrochromic devices and energy storage systems in wearable electronics is highly desirable yet challenging, because self‐powered electrochromic devices often require an open system design for continuous replenishment of the strong oxidants to enable the coloring/bleaching processes. A self‐powered electrochromic device has been developed with a close configuration by integrating a Zn/MnO_2_ ionic battery into the Prussian blue (PB)‐based electrochromic system. Zn and MnO_2_ electrodes, as dual shared electrodes, the former one can reduce the PB electrode to the Prussian white (PW) electrode and serves as the anode in the battery; the latter electrode can oxidize the PW electrode to its initial state and acts as the cathode in the battery. The bleaching/coloring processes are driven by the gradient potential between Zn/PB and PW/MnO_2_ electrodes. The as‐prepared Zn||PB||MnO_2_ system demonstrates superior electrochromic performance, including excellent optical contrast (80.6%), fast self‐bleaching/coloring speed (2.0/3.2 s for bleaching/coloring), and long‐term self‐powered electrochromic cycles. An air‐working Zn||PB||MnO_2_ device is also developed with a 70.3% optical contrast, fast switching speed (2.2/4.8 s for bleaching/coloring), and over 80 self‐bleaching/coloring cycles. Furthermore, the closed nature enables the fabrication of various flexible electrochromic devices, exhibiting great potentials for the next‐generation wearable electrochromic devices.

## Graphical abstract

A self‐powered Zn||PB||MnO_2_ electrochromic device based on a shared electrode design and featuring a closed configuration, has been developed, which consists of a Prussian blue (PB) based electrochromic system and a zinc‐ion battery (ZIB). Both the bleaching and coloring processes are driven by the gradient potentials between Zn/PB and PW/MnO_2_ electrodes instead of external power supply. Owning to the closed nature of Zn||PB||MnO_2_ electrochromic system, a range of integrated flexible electrochromic devices are successfully fabricated, including electrochromic glasses, labels, and wristbands, exhibiting great potentials for the next‐generation wearable electrochromic devices.

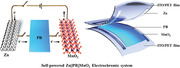

## 1 Introduction

Electrochromic materials (ECMs) can autonomously and reversibly change their optical properties (e.g., transmittance, reflectance, and absorbance) when a short voltage is applied.^[^ ^1^, ^2^, ^3^ ^]^ Due to the unique feature of rapid switchability in bleached/colored state and excellent bistability, they have been utilized in various new applications including wearable and display electronics,^[^ ^4^, ^5^, ^6^ ^]^ while their bleaching/coloring process are strongly relied on the external power sources to drive the electrochromic layer to undergo oxidation/reduction reaction or ion insertion/extraction, restricting the independent and sustainable operation of electrochromic devices. To solve this problem, energy harvesting devices, such as triboelectric nanogenerators (TENGs)^[^ ^7^, ^8^, ^9^ ^]^ and solar panels^[^ ^10^, ^11^, ^12^ ^]^ are introduced to enable self‐power of the electrochromic devices. However, they are environmentally dependent, TENGs need to absorb or converse weak mechanical energy (e.g., wind and raindrops) and solar panels cannot work in rainy/hazy weather or at night, resulting in an unsatisfied controllability. Another approach is to integrate with the energy storage devices,^[^ ^13^, ^14^ ^]^ such as lithium‐ion and sodium‐ion batteries, which can be connected to electrochromic devices to enable short‐term operation within the capacity range. Nevertheless, concerns have arisen regarding safety issues, such as the leakage of organic electrolytes, particularly when deployed in flexible or wearable electrochromic electronics.^[^ ^15^, ^16^ ^]^

To achieve stable self‐powered electrochromic devices, ECMs can be introduced inside the energy storage devices and serve as the cathode,^[^ ^17^, ^18^ ^]^ while the active metals (Mg/Al/Zn) acts as the anode, forming the electrochromic ionic batteries, such as Zn‐WO_3_,^[^ ^19^ ^]^ Zn‐polyaniline (PANI),^[^ ^20^ ^]^ Al‐Prussian blue (PB),^[^ ^21^ ^]^ Al‐W_18_O_49_,^[^ ^22^ ^]^ and Mg‐PB.^[^ ^23^ ^]^ This design successfully combines electrochromism and energy storage functions, the presence of active metals can lead to the reduction of electrochromic materials driven by the potential difference between two electrodes, resulting in the change of color and transmittance of ECMs. To revert to its initial state, these batteries must be connected to an external power supply and subjected to charging. As a result, these two‐electrode electrochromic systems can only achieve a unidirectional self‐powered state change.^[^ ^24^ ^]^

Reversible self‐powered bleaching and coloring cycles in electrochromic devices can be achieved by introducing an additional electrode with high oxidizing ability, resulting in the establishment of a three‐electrode system. ECMs, possess the middle potential between the active metals and oxidizing agents, alternately connect either of them, resulting in the reversible self‐bleaching/coloring electrochromic cycles. Specifically, active metals with the lowest potential are capable of effectively reducing ECMs. Subsequently, the electrodes with high oxidizing ability oxidize the ECMs back to their original state. Strong oxidizing agents, such as O_2_,^[^ ^21^, ^25^ ^]^ H_2_O_2_,^[^ ^26^ ^]^ NaClO,^[^ ^23^ ^]^ ammonium persulfate,^[^ ^27^ ^]^ or etched carbon paper^[^ ^24^ ^]^ have been introduced into electrochromic systems to develop reversible self‐powered electrochromic devices. However, the oxidizing materials in these systems are continuously consumed, requiring open electrochromic systems to realize oxidants replenishment. Nevertheless, the open system configuration leads to electrolyte leakage and continuous volatilization,^[^ ^28^, ^29^ ^]^ rendering the practical application of electrochromic devices. Therefore, it is highly desired for the development of self‐powered electrochromic devices capable of functioning within closed systems, particularly for wearable and portable applications.

**Figure 1:**
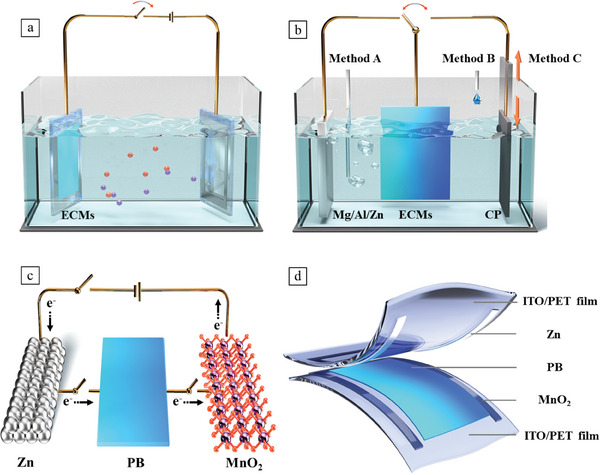
Working principle comparison of the electrochromic systems and the configuration of the flexible electrochromic devices. a) Conventional electrochromic systems, their bleaching/coloring process are strongly relied on the external bias to drive the electrochromic layer to undergo oxidation/reduction reaction or ion insertion/extraction; b) Schematic of the three‐electrode electrochromic systems. The ECMs can be reduced by active metals and undergo changes in color and transmittance, while to revert to its initial state, a series of strong oxidants are used, including O_2_ (Method A), H_2_O_2,_ and NaClO (Method B), and etched carbon paper (Method C); c) Electrochromic mechanism of the Zn||PB||MnO_2_ system, the Zn electrode is capable of reducing the PB electrode to transparent PW when connects the two electrodes, on the other hand, the MnO_2_ electrode can rapidly oxidize the PW electrode to the colored PB electrode. A zinc‐ion battery can be easily established and subjected to charging when the Zn and MnO_2_ electrodes are connected, enabling the continuous, independent, and sustainable operation of electrochromic devices; d) Schematic of the flexible Zn||PB||MnO_2_ electrochromic devices with a closed system design.

Herein, we have developed a self‐powered Zn||PB||MnO_2_ electrochromic device featuring a close configuration, which consists of a PB‐based electrochromic system and a zinc‐ion battery. The Zn and MnO_2_ electrodes, as dual shared electrodes, simultaneously shared by the electrochromic system and a zinc‐ion battery. The Zn electrode possesses the lowest potential, capable of reducing the blue PB to the transparent PW electrode, thus realizing the self‐bleaching process, it also serves as the anode of the zinc‐ion battery. The MnO_2_ electrode, has the highest potential, enabling the oxidization of the PW electrode to its initial PB state, which also acts as the cathode in the zinc‐ion battery. For the PB electrode and its reduced PW state, their potentials always fall between the Zn and MnO_2_ electrodes (Zn < PW < PB < MnO_2_), generating dual gradient potentials between Zn/PB and PW/MnO_2_ electrodes, which drive the self‐bleaching/coloring processes. The as‐prepared Zn||PB||MnO_2_ electrochromic system demonstrates superior electrochromic performance, including an ultra‐high optical contrast (80.6%), rapid bleaching/coloring speed (bleaching/coloring time, denoted as *T*_b_ and *T*_c_,*T*_b_ = 2.0 s, *T*_c_ = 3.2 s). A zinc‐ion battery can be easily obtained and subjected to charging when connects the Zn and MnO_2_ electrodes, enabling the continuous, independent, and sustainable operation of devices for more than 5000 electrochromic cycles, demonstrating outstanding cycling capability. In addition, owning to the closed nature of Zn||PB||MnO_2_ electrochromic system, a range of integrated flexible electrochromic devices were successfully fabricated (**Figure** **1**d), including electrochromic glasses, labels, and wristband, exhibiting great potentials for the next‐generation wearable electrochromic devices.

## 2 Results and Discussion

### 2.1 Preparation and Characterization of MnO_2_ and PB electrodes

**Figure 2:**
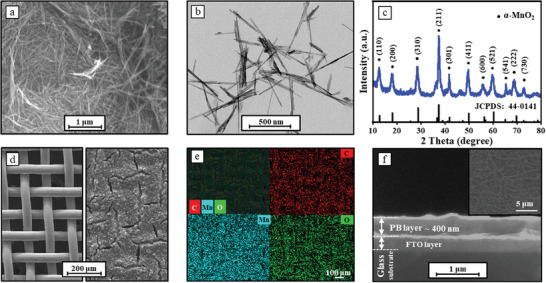
Characterization of the MnO_2_ and PB electrodes. a) SEM and b) TEM images of α‐MnO_2_ power; c) the XRD pattern of the synthesized α‐MnO_2_ power; d) SEM images of the pristine stainless‐steel mesh (left) and coated with MnO_2_ electrode slurry (right); e) The SEM image and the corresponding EDS mapping of the MnO_2_ electrode; f) The surface and lateral SEM images of the PB electrode coated on the FTO glass substrate.

Among the cathode of aqueous rechargeable zinc batteries, Manganese‐based compounds, as one of the most prevalent materials due to their safety, affordability, eco‐friendliness, a high average discharge voltage (1.2–1.4 V versus Zn^2+/^Zn), and a desirable discharge capacity, thus we chose it as the highest potential electrode in our self‐powered electrochromic system. The MnO_2_ powder is synthesized via a hydrothermal method,^[^ ^30^ ^]^ and **Figure** **2**a shows its homogeneous 1D nanofiber structure, the Transmission Electron Microscope (TEM) image (Figure 2b) further confirms its length of a few micrometers with a diameter of ≈50 nm. The high‐resolution (HR‐TEM) image of MnO_2_ (Figure S1, Supporting Information) exhibits distinct lattice fringes with a lattice spacing of 2.41 Å corresponding to the (211) plane, and the selected area electron diffraction (SAED) in Figure S1f (Supporting Information) remarkably reveals the persistence of the (211) crystal plane. The X‐ray diffractometer (XRD) pattern in Figure 2c demonstrates the crystalline phase of α‐MnO_2_ (JCPDS: 44–0141).^[^ ^31^ ^]^ The image on the left in Figure 2d depicts a pristine stainless‐steel mesh chosen as the substrate. The MnO_2_ electrode was prepared by brushing a MnO_2_ slurry onto the stainless‐steel mesh with a loading of 5 mg·cm^−2^. The energy dispersive spectrum (EDS) mapping in Figure 2e confirms the uniform distribution of MnO_2_ powder on the stainless‐steel mesh. For the PB electrode, the PB electrochromic layer was electrodeposited on fluorine‐doped tin oxide (FTO) glass substrates by a three‐electrode method.^[^ ^32^ ^]^ After evaluating several electrodeposition schemes, the optimum electrodeposition current density and time were determined to be −15 µA cm^−2^ and 10 min, respectively (Figure S2, Supporting Information). Figure S3 (Supporting Information) illustrates the PB electrode's rough surface, marked by numerous ravines, likely arising from internal stress discrepancies inherent in the electrochemical deposition process. After deposition and removal of the PB‐FTO glass from the electrodeposition solution, the PB layer undergoes stress imbalance, leading to crack formation. In the XRD pattern of the PB/FTO glass (Figure S4a, Supporting Information), two new peaks appear at 17.5°and 24.8°, corresponding to the (200) and (220) diffraction peaks of PB (JCPDS: 01–0239), respectively. The thickness of as‐obtained PB film is measured to be 300 nm through SEM analysis (Figure 2f), EDS mapping reveals that the compositional distributions of the four elements (K, Fe, C, and N) in the PB electrochromic layer (Figures S3 and S4, Supporting Information).

### 2.2 Electrochromic Mechanism of Zn||PB||MnO_2_ System

**Figure 3:**
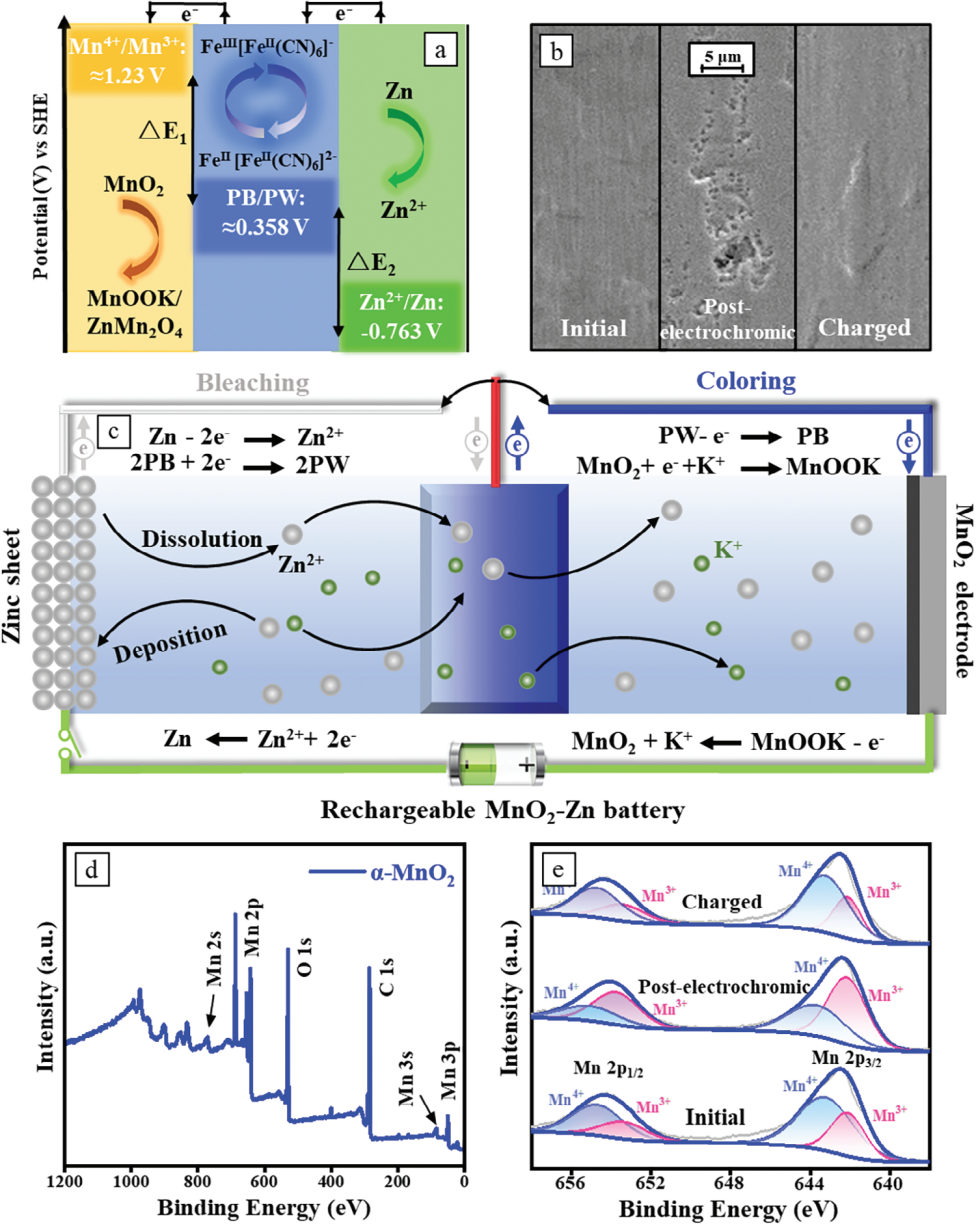
Electrochromic mechanism of the Zn||PB||MnO_2_ system. a) Illustration of the flow of electrons during the bleaching/coloring process and the standard electrode potential of the Zn, PB and MnO_2_ electrodes; b) The SEM images of the pristine, post‐electrochromic, and recharged Zn electrode; c) Schematic diagram of the charging of the MnO_2_ electrode; d) XPS spectrum of the MnO_2_ electrode; e) Mn 2p XPS spectra of MnO_2_ electrode at initial, post‐electrochromic, and recharged states.

**Figure** **3**a illustrates the configuration of the Zn||PB||MnO_2_ three‐electrode system, with the solution containing Zn^2+^, Mn^2+^, and K^+^ serving as the electrolyte. The standard electrode potential of PB/PW and Zn^2+^/Zn and is 0.358 and −0.763 V^[^ ^24^ ^]^ versus standard hydrogen electrode (SHE), respectively. Electrons flow from the Zn electrode to the PB electrode, reducing Fe^3+^ to Fe^2+^ upon connecting the two electrodes, leading to the transformation of the blue PB electrode to the colorless PW electrode. The MnO_2_ electrode has a standard electrode potential of 1.23 V^[^ ^33^ ^]^ versus SHE, electrons flow from PW electrode to MnO_2_ electrode, the Zn^2+^/K^+^ inserted PW will extract and lead to blue PB (Figure S5, Supporting Information). Both the bleaching and coloring processes are driven by the potential difference between Zn/PB electrodes and PW/MnO_2_ electrodes, as confirmed by their open‐circuit voltage (Figure S6, Supporting Information). The mechanism can be further expressed by the following reactions:

#### 2.2.1 Bleaching Process

(1) $$\mathrm{Zn}-2e^{-}\to Zn^{2+}$$

(2) $$\mathrm{KF}e^{\mathrm{III}}\left[{\mathrm{Fe}}^{\mathrm{II}}{\left(\mathrm{CN}\right)}_{6}\right]\left(\mathrm{PB}\right)+K^{+}+e^{-}\to K_{2}{\mathrm{Fe}}^{\mathrm{II}}\left[{\mathrm{Fe}}^{\mathrm{II}}{\left(\mathrm{CN}\right)}_{6}\right]\left(\mathrm{PW}\right)$$

#### 2.2.2 Coloring Process

(3) $$\mathrm{Mn}O_{2}+e^{-}+K^{+}\to \mathrm{MnOOK}$$

(4) $$K_{2}{\mathrm{Fe}}^{\mathrm{ii}}\left[{\mathrm{Fe}}^{\mathrm{ii}}{\left(\mathrm{CN}\right)}_{6}\right]\left(\mathrm{PW}\right)-e^{-}\to \mathrm{KF}e^{\mathrm{iii}}\left[{\mathrm{Fe}}^{\mathrm{ii}}{\left(\mathrm{CN}\right)}_{6}\right]\left(\mathrm{PB}\right)+K^{+}$$

Figure 3b depicts the pristine zinc electrode in its initial state, exhibiting a smooth appearance. Following more than 100 cycles of coloring/bleaching switching, the zinc electrode displayed significant corrosion. The corrosion can be attributed to the continuous dissolution of the active metals (Mg/Zn/Al) electrode when connected to the ECMs to drive the coloring/bleaching process, the active metals continually dissolve.^[^ ^22^, ^34^ ^]^ Consequently, there is a continuous rise of the corresponding metal‐positive ions in the electrolyte, and even leading to the rapid degradation of the ECMs performance and a reduction in the stability and lifespan of the devices.^[^ ^32^ ^]^ This phenomenon is common and inevitable in the self‐powered system that utilizing active metals to reduce the ECMs. In contrast, our Zn||PB||MnO_2_ system presents a promising solution to alleviate the dissolution and consumption of zinc. By connecting Zn electrode to the MnO_2_ electrode, a Zn/MnO_2_ battery is formed and subjected to charging, therefore, the dissolved zinc ions undergo reduction, leading to their re‐deposition on the zinc surface, effectively restoring its smoothness. When the MnO_2_ electrode connects to the PW electrode, PW undergoes oxidation, leading to the embedding of K^+^ and Zn^2+^ ions within the MnO_2_ structure, forming MnOOK/ZnMn_2_O_4_^[^ ^35^ ^]^ and reducing the potential of the MnO_2_ electrode. As depicted in Figure S7 (Supporting Information), the potential of the MnO_2_ electrode gradually decreased with increasing cycle number, and the electrochromic switching speed undergone a decrease, and the transmittance of PB in the colored state was not as high as it was initially. By the 120^th^ cycle, the potential of the MnO_2_ electrode dropped to 1.28 V (vs Zn^2+^/Zn). Fortunately, a Zn/MnO_2_ ionic battery can be established when the two electrodes are connected and charged to extract K and Zn ions from MnOOK/ZnMn_2_O_4_ (Figure S8, Supporting Information), restoring the potential of the MnO_2_ electrode and ensuring sustainable and rapid electrochromic cycles. The EDS images of the post‐electrochromic/charged MnO_2_ electrode further confirmed our assumption. The results revealed a significant presence of Zn and K elements, and after charging, most Zn elements and all K elements disappeared. The reaction of the charging process can be explained by the following mechanism (Figure 3c):

#### 2.2.3 Cathode Reaction

(5) $$\mathrm{MnOOK}-e^{-}↔K^{+}+\mathrm{Mn}O_{2}$$

#### 2.2.4 Anode Reaction

(6) $$Zn^{2+}+2e^{-}↔\mathrm{Zn}$$

X‐ray photoelectron spectroscopy (XPS) is further employed to detect the composition of the MnO_2_ electrode, and the results are shown in Figure 3d. The Mn 2p spectrum (Figure 3e) reveals two peaks positioned at 642.6 and 654.3 eV, corresponding to the binding energies of Mn 2p_3/2_ and 2p_1/2_,^[^ ^36^ ^]^ respectively. Both the Mn 2p peaks shift to  low binding energy when the MnO_2_ cathode is at the fully discharged state due to the electrochromic cycles, corresponding to the reduction of manganese in MnO_2_. Then these peaks return to the high binding energy since the manganese in MnO_2_ is oxidized to the initial state at the fully charged state.

### 2.3 Electrochromic Performance of the Zn||PB||MnO_2_ System

**Figure 4:**
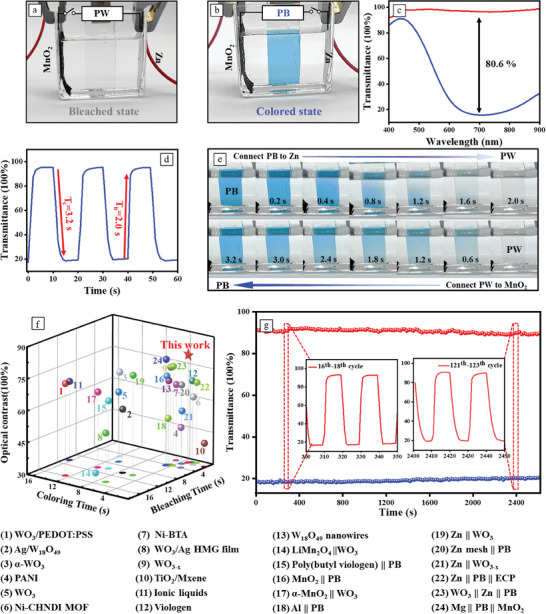
a,b) Optical photos of bleached state and colored state of PB electrode; c) UV–vis transparence spectra of PB and PW at a wavelength from 400 to 900 nm (colored and bleached states); d) In situ transmittance measurement at 700 nm of PB connected with Zn electrode and MnO_2_ electrode for 10 s, respectively; e) Photographs of the corresponding bleaching/coloring process, when connected with the Zn electrode and the MnO_2_ electrode, respectively; f) Optical contrast and switching time comparison of the Zn||PB||MnO_2_ system to previously reported electrochromic systems; g) In situ transmittance measurement of Zn||PB||MnO_2_ system under repeated bleaching and coloring cycles, the switching time is set to 10 s.

We further characterize the electrochromic performance of the as‐prepared Zn||PB||MnO_2_ system via Ultraviolet‐Visible (UV–vis) spectroscopy. **Figure** **4**a,b displays optical photos of the PB electrode in its colored and bleached states, respectively. The optical modulation (Figure 4c) between these two states exceeds 80% at 700 nm, increasing from 16.0% to 96.6%. Owning to the large potential difference, the electrochromic system demonstrated ultra‐fast electrical response speed (Movie S1, Supporting Information). In‐situ dynamic optical transmittance measurements (Figure 4d) were performed at a wavelength of 700 nm by connecting the PB electrode to the Zn electrode (bleaching) or MnO_2_ electrode (coloring) in turn and repeatedly. The transmittance jumps from 19.96% to 92.0% in just 2.0 s when the Zn electrode is linked to PB electrode (with a size of 2 × 3 cm^2^) and decrease from 94.6% to 22.6% in 3.2 s when connecting to MnO_2_ electrodes. The rapid electrochromic switching rate is also attributable to the optimization of the electrolyte. Given the incorporation of both Zn and MnO_2_ electrodes within the electrochromic device, creating an all‐in‐one configuration, the electrolyte is consequently shared between the electrochromic system and the Zn/MnO_2_ battery. A mixture of ZnSO_4_ and MnSO_4_ was initially chosen, which was commonly used in Zn/MnO_2_ batteries, with the addition of MnSO_4_ aimed at enhancing the stability of the MnO_2_ electrode. However, it exhibited a slower bleaching/coloring rate and low optical contrast, which could be attributed to the larger ionic radius of Zn^2+^, making it less prone to intercalation and deintercalation within the PB electrode. Therefore, we attempted to introduce the smaller radius of K^+^ ions, resulting in a significant improvement in the switching rate (Figure S9, Supporting Information). The Zn^2+^/K^+^ electrolyte leads to the sharpest CV peaks and one pair of well‐defined redox peaks, suggesting a rapid K^+^ insertion/extraction kinetics, which can be attribute to the suitable size of hydrate K^+^. In contrast, the CV curve of the PB film in 2 m ZnSO_4_ displays broad and multi‐redox peaks, indicating the slow Zn^2+^ insertion/extraction speed. The photographs of the corresponding bleaching/coloring processes are shown in Figure 4e. Optical contrast and switching speed are the important indexes to evaluate the properties of the electrochromic devices, compared with the recently reported devices that based on a single electrochromic electrode (e.g., WO_3_, W_17_O_47_, W_18_O_49_, TiO_2_, PANI, Viologen),^[^ ^37^, ^38^, ^39^, ^40^, ^41^, ^42^, ^43^, ^44^, ^45^, ^46^, ^47^, ^48^, ^49^, ^50^, ^51^ ^]^ two‐electrode configurations (e.g., MnO_2_||WO_3_, NiO||WO_3_, Al||PB, Zn||WO_3_, Zn||WO_3_),^[^ ^5^, ^19^, ^21^, ^32^, ^52^, ^53^, ^54^, ^55^ ^]^ and self‐powered three‐electrode configurations (e.g., WO_3_||Zn||PB, Mg||PB||MnO_2_),^[^ ^24^, ^56^, ^57^ ^]^ an unprecedented combination of high optical contrast (80.6%) and fast switching speed (*T*_b_ = 2.0 s, *T*_c_ = 3.2 s) could be achieved by our Zn||PB||MnO_2_ system (Figure 4f; Table S1, Supporting Information). Even though several electrochromic devices with rapid bleaching and coloring speeds have been developed, they are limited to low optical contrast. A real air‐working Zn||PB||MnO_2_ electrochromic system was also developed and demonstrated similar optical contrast and switching speed, while its cyclic performance undergone a slightly decrease, which could attribute to the limited area of the MnO_2_ electrode (Figure S10, Supporting Information). Additionally, we assessed the cycle stability of the Zn||PB||MnO_2_ system, successfully extending the electrochromic duration to 2600 s (130 cycles) without significant degradation in electrochromic performance (Figure 4g), representing the outstanding cyclic performance among the reported self‐powered electrochromic systems. Consequently, after the electrochromic cycles, we connected the Zn electrode to the MnO_2_ electrode, forming a zinc‐ion battery and subjecting to charging, continued to undergo electrochromic cycles. After repeating these procedures 50 times (>5000 cycles), the Zn||PB||MnO_2_ system was still capable of enduring more than 110 self‐powered bleaching/coloring cycles (Figure S11, Supporting Information). The slight reduction in the number of cycles might be attributed to the capacity decay of the Zn/MnO_2_ battery. Initially, the battery displayed a high capacity of 300.9 mA h g^−1^ at 0.1 A g^−1^, after 50 cycles, the capacity declined to 241.2 mA h g^−1^ with a retention of 80.1% (Figure S12d, Supporting Information). The overall electrochemical performance of the Zn/MnO_2_ cell are further investigated. Figure S12b,c, Supporting Information) displays the rate capability and corresponding charge/discharge profiles, illustrating capacities of 308.8, 280, 237.5, 173.1, and 116.7 mAh g^−1^ at 0.1, 0.2, 0.5, 1, and 2C, respectively. Additionally, the cyclic performance of the Zn/MnO_2_ cell at 1 A g^−1^ is measured, exhibiting a high initial capacity of 203.68 mA h g^−1^ and retaining 79.8% of its initial capacity after 500 cycles. Moreover, Figure S13 (Supporting Information) illustrates that Zn, MnO_2_, and PB electrodes, following thousands of electrochromic cycles, largely maintain their original microstructures.

### 2.4 Flexible Electrochromic Wearable Device

**Figure 5:**
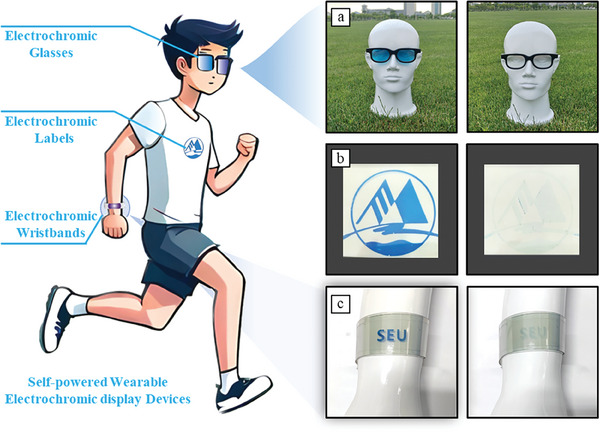
Illustration of the wearable electrochromic devices, including electrochromic smart glasses, labels, and wristbands. Smart glasses a) in the colored and bleached state; b) A smart electrochromic label (electrochromic area: 5 cm × 5 cm) in the colored and bleached state; c) A wearable wristband (20 cm × 3.5 cm) assembled with Zn||PB||MnO_2_ electrochromic system in the colored and the bleached state.

A key feature of the Zn||PB||MnO_2_ system is its ability to operate within a closed environment, marking the first instance of self‐powered system, which enables wearable electrochromic devices. We have utilized an improved gradient potential‐driven deposition method to deposit PB deposition on the flexible ITO/PET substrate^[^ ^55^ ^]^ (Figure S14, Supporting Information), demonstrating better adhesion to the substrate rather than electrodeposition, and fabricated a series of electrochromic devices, including smart glasses, labels, and wristbands. The electrochromic glasses can serve as anti‐glare devices in their colored state, safeguarding the eyes from intense light exposure. **Figure** **5**a demonstrates the coloring state and bleaching state of the glasses, exhibiting a significant difference in color and transmittance (Figure S15 and Movie S2, Supporting Information). The transition between the two states was rapid, with bleaching taking 4 s and coloring taking 10 s. Additionally, we prepared an electrochromic label (Figure 5b) that can be easily attached to clothing, providing an intelligent display function based on personal preference (Movie S3, Supporting Information) for conventional attire. Moreover, it can still operate normally at ice/hot water and even fold to 180°, verifying its outstanding electrochromic stability and flexibility (Figure S16, Supporting Information). Figure 5c illustrates the wearable wristband (20 cm × 3.5 cm) integrated with the Zn||PB||MnO_2_ electrochromic system, showing both the colored and bleached states. The electrochromic performance of the wristband remains unaffected even when bent at angles exceeding 90° (Movie S4, Supporting Information).

## 3 Conclusion

In summary, we have developed a self‐powered electrochromic system with a closed configuration, which consists of a PB‐based electrochromic system and a zinc‐ion battery based on the shared electrodes (Zn and MnO_2_ electrodes) design. The as‐prepared Zn||PB||MnO_2_ electrochromic system exhibits outstanding electrochromic performance, including an ultra‐high optical contrast (80.6%), rapid bleaching/coloring speed (*T*_b_ = 2.0 s, *T*_c_ = 3.2 s). Impressively, the as‐prepared electrochromic device can undergo more than 130 electrochromic cycles without external power supply, the introduced Zn/MnO_2_ zinc‐ion battery can be subjected to charging, enabling the continuous, independent, and sustainable operation of the electrochromic device for more than 5000 electrochromic cycles, demonstrating its excellent cycling capability. In addition, due to the closed nature of the Zn||PB||MnO_2_ electrochromic system, we successfully fabricated a series of wearable electrochromic devices, such as smart glasses, labels and wristbands, demonstrating their great potentials for the next‐generation wearable electrochromic devices.

## 4 Experimental Section

### Materials

Potassium ferricyanide (K_3_[Fe(CN)_6_], 99%), iron chloride (FeCl_3_, 98%), potassium chloride (KCl, 99%), zinc sulfate heptahydrate (ZnSO_4_·7H_2_O, 99%), potassium sulfate (K_2_SO_4_, 99%), manganese sulfate monohydrate (MnSO_4_·H_2_O, 99%), ammonium sulfate ((NH_4_)_2_SO_4_, 99%), ammonium persulfate ((NH_4_)_2_S_2_O_8_, 98%) and N‐methylpyrrolidone (NMP, 98%) were purchased from Aladdin (Shanghai, China) and used without further purification. Zn foil (Zn, 99.99%), Ni sheet (99.99%), and stainless‐steel wire mesh were purchased from Tianjin Aiwixin Chemical Technology Co. Ltd. Super‐P conductor and polyvinylidene difluoride (PVDF) were purchased from Nanjing Moges Energy Technology Co. Ltd. Fluorine‐doped tin oxide (FTO) glass and indium tin oxide deposited on PET substrate (ITO/PET) were purchased from Zhuhai Kaiwei Optoelectronics Technology Co. Ltd. Deionized water was used for all experiments.

### Preparation of α‐MnO_2_ and MnO_2_ Electrode

Typically, 0.2 m MnSO_4_·H_2_O, 0.2 m (NH_4_)_2_S_2_O_8_, and 0.8 m (NH_4_)_2_SO_4_ were dissolved in deionized water to obtain a 40 mL aqueous solution. Then, the mixed solution was transferred into an autoclave and maintained at 140 °C for 12 h. The obtained product of α‐MnO_2_ was washed with distilled water and ethanol several times, and dried in a vacuum oven at 80 °C overnight. The MnO_2_ electrode was prepared by mixing the active material α‐MnO_2_ power (70 wt.%), super‐P conductor (20 wt.%), and polyvinylidene fluoride (PVDF, 10 wt.%) in N‐methylpyrrolidone (NMP) to form a homogeneous slurry. Then, the slurry was coated onto stainless steel meshes, followed by vacuum drying at 70 °C for ≈12 h. The mass loading of the active material was ≈5 mg cm^−2^.

### Deposition of PB Electrodes

The PB electrochromic layer was electrodeposited on fluorine‐doped tin oxide (FTO) glass substrate (14–16‐ohm sq^−1^) by a three‐electrode method, with carbon cloth and Ag/AgCl as counter and reference electrodes, respectively. The aqueous solution for electrodeposition was prepared according to the previous reports. In a typical procedure, PB film was electrodeposited on the FTO glass by applying a constant current density (−15 µA cm^−2^) for 10 min in an aqueous electrolyte containing 5 mm FeCl_3_, 5 mm K_3_[Fe(CN)_6_], and 200 mM KCl. The PB film was deposited onto an indium tin oxide (ITO)‐coated PET substrate (≈10 ohms sq^−1^) according to a modified galvanic‐driven deposition method. Briefly, A piece of metallic nickel was tightly attached to one side of the ITO/PET substrate through a conductive double‐sided tape and then immersed into a homogeneous solution containing 10 mm K_3_[Fe(CN)_6_], 10 mm FeCl_3,_ and 50 mm KCl for ≈120 s. Then the PB/ITO electrode was flushed with deionized water and dried at 70 °C.

### Assembly of the Flexible Electrochromic Devices

The electrochromic smart glasses consisted of a deposited PB film coated on ITO/PET substrate and a Zn and an MnO_2_ electrode frame placed on the edge. Zn and MnO_2_ electrode connected with the ITO/PET substrate indirectly, scotch tapes acted as a spacer to avoid short‐circuiting between different electrodes. A 1 mm thick 3 m waterproof tape was used between the two ITO/PET substrates to bond, and the homogeneous solution of 2 m ZnSO_4_, 0.1 m MnSO_4_, and 0.1 m K_2_SO_4_ served as the electrolyte. In addition, AB glue was employed to seal the sides of the device to prevent leakage. The preparation of labels and wristbands were similar to that of smart glasses. The pattern and the logo of SEU were adhered to the ITO/PET substrate, and the template were removed after being deposited.

### Material Characterizations

The phase of α‐MnO_2_ was analyzed by a Haoyuan DX‐2700BH powder X‐ray diffractometer using Cu‐K𝛼 radiation (𝜆 = 1.5406 Å) with MDI Jade software. The morphology and structure of the samples were measured by scanning electron microscopy (SEM, FEI Sirion) with 20 KV working voltage. Transmission electron microscope (TEM) was performed on a Thermo Scientific Talos F200X and the working voltage was set as 20 KV. X‐ray Photoelectron Spectroscopy (XPS, Thermo Scientific K‐Alpha) was conducted to study the surface composition and electronic states of the MnO_2_ electrode. The purchased zinc flakes underwent sequential ultrasonic cleaning with alcohol and deionized water, followed by drying. Subsequently, for both the post‐electrochromic (discharged) and charged Zn electrodes, they were rinsed with deionized water and left to air dry in a fume hood for further characterization.

### Property Characterization

A UV–vis spectrophotometer (UV‐2600, Shimadzu) was used to characterize the specular transmittance of the sample in the range of 400–900 nm. The electrochemical performance of the Zn||PB||MnO_2_ EC system was characterized on an electrochemical workstation (Biologic workstation SP150). Galvanostatic discharge/charge cycling measurements were performed with a multi‐channel battery test system (Newarwe, Shenzhen, China). The potential change of MnO_2_ electrode during EC cycles was measured using an Ag/AgCl electrode (saturated KCl) as the negative reference electrode. The purchased zinc flakes underwent sequential ultrasonic cleaning with alcohol and deionized water, followed by drying. Subsequently, for both the post‐electrochromic (discharged) and charged Zn electrodes, they were rinsed with deionized water and left to air dry in a fume hood for further characterization. The transmittance of the FTO glass or ITO/PET was used as the baseline during the measurement.

## Conflict of Interest

The authors declare no conflict of interest.

## Supporting information

Supporting Information

Supplemental Movie1

Supplemental Movie2

Supplemental Movie3

Supplemental Movie4

Supplemental Movie5

Supplemental Movie6

## Data Availability

The data that support the findings of this study are available in the supplementary material of this article.

## References

[1] G. Yang , Y.‐M. Zhang , Y. Cai , B. Yang , C. Gu , S. X.‐A. Zhang , Chem. Soc. Rev. 2020, 49, 8687. 33078186 10.1039/d0cs00317d

[2] Z. Wang , X. Wang , S. Cong , F. Geng , Z. Zhao , Mater. Sci. Eng. R Rep. 2020, 140, 100524.

[3] Q. Zhao , J. Wang , X. Ai , Z. Pan , F. Xu , J. Wang , Y. Gao , Nano Energy 2021, 89, 106356.

[4] J. Li , P. Yang , X. Li , C. Jiang , J. Yun , W. Yan , K. Liu , H. J. Fan , S. W. Lee , ACS Energy Lett. 2022, 8, 1.

[5] C. Gu , A.‐B. Jia , Y.‐M. Zhang , S. X.‐A. Zhang , Chem. Rev. 2022, 122, 14679. 35980039 10.1021/acs.chemrev.1c01055 PMC9523732

[6] R. T. Ginting , M. M. Ovhal , J.‐W. Kang , Nano Energy 2018, 53, 650.

[7] M.‐H. Yeh , L. Lin , P.‐K. Yang , Z. L. Wang , ACS Nano 2015, 9, 4757. 25808880 10.1021/acsnano.5b00706

[8] J. Wang , C. Meng , Q. Gu , M. C. Tseng , S. T. Tang , H. S. Kwok , J. Cheng , Y. Zi , ACS Nano 2020, 14, 3630. 32078294 10.1021/acsnano.0c00107

[9] L. Zhou , D. Liu , L. Liu , L. He , X. Cao , J. Wang , Z. L. Wang , Research 2021, 2021, 4673028. 33796860 10.34133/2021/4673028 PMC7982057

[10] N. C. Davy , M. Sezen‐Edmonds , J. Gao , X. Lin , A. Liu , N. Yao , A. Kahn , Y.‐L. Loo , Nat. Energy. 2017, 2, 17104.

[11] H. Ling , J. Wu , F. Su , Y. Tian , Y. J. Liu , Nat. Commun. 2021, 12, 1010. 33579925 10.1038/s41467-021-21086-7 PMC7881180

[12] H. Zhang , F. Sun , J. Feng , H. Ling , D. Zhou , G. Cao , S. Wang , F. Su , Y. Tian , Y. Tian , Cell Rep. Phys. Sci. 2022, 3, 101193.

[13] F. Zhao , B. Wang , W. Zhang , S. Cao , L. Liu , A. Y. Elezzabi , H. Li , W. W. Yu , Mater. Today. 2023, 66, 431.

[14] J. Chen , G. Adit , L. Li , Y. Zhang , D. H. Chua , P. S. Lee , Energy Environ. Mater. 2023, 6, e12633.

[15] K. Liu , Y. Liu , D. Lin , A. Pei , Y. Cui , Sci. Adv. 2018, 4, eaas9820. 29942858 10.1126/sciadv.aas9820 PMC6014713

[16] Q. Wang , L. Jiang , Y. Yu , J. Sun , Nano Energy 2019, 55, 93.

[17] S. Kandpal , T. Ghosh , C. Rani , A. Chaudhary , J. Park , P. S. Lee , R. Kumar , ACS Energy Lett. 2023, 8, 1870.

[18] Q. Zhao , Z. Pan , B. Liu , C. Bao , X. Liu , J. Sun , S. Xie , Q. Wang , J. Wang , Y. Gao , Nano‐Micro Lett. 2023, 15, 87. 10.1007/s40820-023-01056-y PMC10082149 37029252

[19] H. Li , C. J. Firby , A. Y. Elezzabi , Joule 2019, 3, 2268.

[20] Y. Wang , H. Jiang , R. Zheng , J. Pan , J. Niu , X. Zou , C. Jia , J. Mater. Chem. A 2020, 8, 12799.

[21] J. Wang , L. Zhang , L. Yu , Z. Jiao , H. Xie , X. W. Lou , X. Wei Sun , Nat. Commun. 2014, 5, 4921. 25247385 10.1038/ncomms5921

[22] J.‐L. Wang , S.‐Z. Sheng , Z. He , R. Wang , Z. Pan , H.‐Y. Zhao , J.‐W. Liu , S.‐H. Yu , Nano Lett. 2021, 21, 9976. 34813332 10.1021/acs.nanolett.1c03438

[23] H. Zhang , Y. Yu , L. Zhang , Y. Zhai , S. Dong , Chem. Sci. 2016, 7, 6721. 28451116 10.1039/c6sc02347a PMC5355813

[24] Y. Luo , H. Jin , Y. Lu , Z. Zhu , S. Dai , L. Huang , X. Zhuang , K. Liu , L. Huang , ACS Energy Lett. 2022, 7, 1880.

[25] W. Wu , W. C. Poh , J. Lv , S. Chen , D. Gao , F. Yu , H. Wang , H. Fang , H. Wang , P. S. Lee , Adv. Energy Mater. 2023, 13, 2204103.

[26] J. Zhao , Y. Tian , Z. Wang , S. Cong , D. Zhou , Q. Zhang , M. Yang , W. Zhang , F. Geng , Z. Zhao , Angew. Chem., Int. Ed. 2016, 55, 7167. 10.1002/anie.201602657 27159245

[27] S. Zhao , X. Gao , L. Chen , W. Huang , Y. Liu , Appl. Mater. Today. 2022, 28, 101543.

[28] Y. Zhou , X. Dong , Y. Mi , F. Fan , Q. Xu , H. Zhao , S. Wang , Y. Long , J. Mater. Chem. A. 2020, 8, 10007.

[29] X. Ren , S. Hu , Z. Jia , T. Qin , Y. Sui , F. Wang , W. Pan , C. Yang , Adv. Funct. Mater. 2022, 32, 2206127.

[30] A. Chen , C. Zhao , J. Gao , Z. Guo , X. Lu , J. Zhang , Z. Liu , M. Wang , N. Liu , L. Fan , Y. Zhang , N. Zhang , Energy Environ. Sci. 2023, 16, 275.

[31] R. Yang , Z. Guo , L. Cai , R. Zhu , Y. Fan , Y. Zhang , P. Han , W. Zhang , X. Zhu , Q. Zhao , Z. Zhu , C. K. Chan , Z. Zeng , Small 2021, 17, 2103052. 10.1002/smll.202103052 34719844

[32] H. Li , W. Zhang , A. Y. Elezzabi , Adv. Mater. 2020, 32, 2003574. 10.1002/adma.202003574 32954551

[33] X. Xiao , Z. Zhang , Y. Wu , J. Xu , X. Gao , R. Xu , W. Huang , Y. Ye , S. T. Oyakhire , P. Zhang , B. Chen , E. Cevik , S. M. Asiri , A. Bozkurt , K. Amine , Y. Cui , Adv. Mater. 2023, 35, 2211555. 10.1002/adma.202211555 37149287

[34] Z. Wu , H. Wang , Q. Ding , K. Tao , W. Shi , C. Liu , J. Chen , J. Wu , Adv. Funct. Mater. 2023, 33, 2300046.

[35] Y. Xu , G. Zhang , J. Liu , J. Zhang , X. Wang , X. Pu , J. Wang , C. Yan , Y. Cao , H. Yang , W. Li , X. Li , Energy Environ. Mater. 2023, 6, e12575.

[36] S. Wang , Z. Yuan , X. Zhang , S. Bi , Z. Zhou , J. Tian , Q. Zhang , Z. Niu , Angew. Chem., Int. Ed. 2021, 60, 7056. 10.1002/anie.202017098 33443304

[37] L. Zhang , D. Chao , P. Yang , L. Weber , J. Li , T. Kraus , H. J. Fan , Adv. Energy Mater. 2020, 10, 2000142.

[38] W. C. Poh , A. L. Eh , W. Wu , X. Guo , P. S. Lee , Adv. Mater. 2022, 34, 2206952. 10.1002/adma.202206952 36255145

[39] T. Li , S. Li , X. Li , Z. Xu , J. Zhao , Y. Shi , Y. Wang , R. Yu , X. Liu , Q. Xu , W. Guo , Sci. Bull. 2020, 65, 225. 10.1016/j.scib.2019.11.028 36659176

[40] Q. Zhao , J. Wang , X. Ai , Y. Duan , Z. Pan , S. Xie , J. Wang , Y. Gao , InfoMat 2022, 4, e12298.

[41] C. Wu , Z. Shao , W. Zhai , X. Zhang , C. Zhang , C. Zhu , Y. Yu , W. Liu , ACS Nano 2022, 16, 2621. 35081308 10.1021/acsnano.1c09234

[42] S.‐Z. Sheng , J.‐L. Wang , B. Zhao , Z. He , X.‐F. Feng , Q.‐G. Shang , C. Chen , G. Pei , J. Zhou , J.‐W. Liu , S.‐H. Yu , Nat. Commun. 2023, 14, 3231. 37270627 10.1038/s41467-023-38353-4 PMC10239468

[43] R. Li , X. Ma , J. Li , J. Cao , H. Gao , T. Li , X. Zhang , L. Wang , Q. Zhang , G. Wang , C. Hou , Y. Li , T. Palacios , Y. Lin , H. Wang , X. Ling , Nat. Commun. 2021, 12, 1587. 33707439 10.1038/s41467-021-21852-7 PMC7952574

[44] Z. Wu , Z. Lian , S. Yan , J. Li , J. Xu , S. Chen , Z. Tang , S.‐P. Wang , K. W. Ng , ACS Nano 2022, 16, 13199. 35938940 10.1021/acsnano.2c06479

[45] X.‐A. Liu , J. Wang , D. Tang , Z. Tong , H. Ji , H.‐Y. Qu , J. Mater. Chem. A 2022, 10, 12643.

[46] C. Su , Z. Zhao , D. He , H. Song , C. Zhao , W. Mai , Nano Energy 2023, 111, 108396.

[47] A. L. Eh , J. Chen , S. H. Yu , G. Thangavel , X. Zhou , G. Cai , S. Li , D. H. Chua , P. S. Lee , Adv. Sci. 2020, 7, 1903198. 10.1002/advs.201903198 PMC7341104 32670746

[48] Q. Wang , S. Cao , Q. Meng , K. Wang , T. Yang , J. Zhao , B. Zou , Mater. Horiz. 2023, 10, 960. 36606592 10.1039/d2mh01365g

[49] J. Feng , Y. Luo , X. Wang , G. Cai , R. Cao , Small 2023, 19, 2304691. 10.1002/smll.202304691 37403296

[50] G. Cai , P. Cui , W. Shi , S. Morris , S. N. Lou , J. Chen , J. Ciou , V. K. Paidi , K. Lee , S. Li , P. S. Lee , Adv. Sci. 2020, 7, 1903109. 10.1002/advs.201903109 PMC7578889 33101842

[51] Z. Wang , X. Jia , P. Zhang , Y. Liu , H. Qi , P. Zhang , U. Kaiser , S. Reineke , R. Dong , X. Feng , Adv. Mater. 2021, 34, 2106073. 10.1002/adma.202106073 PMC11469086 34613639

[52] L. Liu , X. Diao , Z. He , Y. Yi , T. Wang , M. Wang , J. Huang , X. He , X. Zhong , K. Du , Energy Stor. Mater. 2020, 33, 258.

[53] C. Deng , K. Zhang , L. Liu , Z. He , J. Huang , T. Wang , Y. Liu , X. He , K. Du , Y. Yi , J. Mater. Chem. A 2022, 10, 17326.

[54] Y. Li , P. Sun , J. Chen , X. Zha , X. Tang , Z. Chen , Y. Zhang , S. Cong , F. Geng , Z. Zhao , Adv. Mater. 2023, 35, 2300116. 10.1002/adma.202300116 36921294

[55] Y. Ding , H. Sun , Z. Li , C. Jia , X. Ding , C. Li , J.‐G. Wang , Z. Li , J. Mater. Chem. A 2023, 11, 2868.

[56] W. Zhang , H. Li , A. Y. Elezzabi , Adv. Funct. Mater. 2023, 33, 2300155.

[57] Q. Ma , J. Chen , H. Zhang , Y. Su , Y. Jiang , S. Dong , ACS Energy Lett. 2022, 8, 306.

