# Supplementary material for: Potential Gradient‐Driven Dual‐Functional Electrochromic and Electrochemical Device Based on a Shared Electrode Design
Source: Adv Sci (Weinh). 2024 May 20;11(28):2401948. doi: 10.1002/advs.202401948 (PMC11267289; doi:10.1002/advs.202401948)
Supplement: Supplementary file 1 — Supporting Information [file ADVS-11-2401948-s003.pdf]

ADVANCED SCIENCE
Open Access

Supporting Information

for *Adv. Sci.*, DOI 10.1002/advs.202401948

Potential Gradient-Driven Dual-Functional Electrochromic and Electrochemical Device Based on a Shared Electrode Design

Gang Xu, Wei Zhang, Guangjun Zhu, Huan Xia, Hanning Zhang, Qian Xie, Peng Jin, Haoyu Zhang, Chengjie Yi, Ruqian Zhang, Lingfeng Ji, Tao Shui, Nosipho Moloto, Wei She* and ZhengMing Sun**

Supporting Information

Potential Gradient-Driven Dual-Functional Electrochromic and Electrochemical Device based on a Shared Electrode Design

Gang Xu[1†], Wei Zhang[1†]*, Guangjun Zhu[1,2], Huan Xia[1], Hanning Zhang[1], Qian Xie[1], Peng Jin[3], Haoyu Zhang[1], Chengjie Yi[1], Ruqian Zhang[1], Lingfeng Ji[1], Tao Shui[1], Nosipho Moloto[4], Wei She[1,2]*, ZhengMing Sun[1]*

[1]Jiangsu Key Laboratory of Advanced Metallic Materials, School of Materials Science and Engineering, Southeast University, Nanjing, 211189, China.

[2]State Key Laboratory of High Performance Civil Engineering Materials, Southeast University, Nanjing, 211189, China.

[3]Department of Civil and Mechanical Engineering, Technical University of Denmark, 2800 Kgs, Lyngby, Denmark.

[4]Molecular Science Institute, School of Chemistry, University of the Witwatersrand, Private Bag 3, Wits2050, South Africa.

[†]These authors contributed equally to this work.

*Corresponding authors: w69zhang@seu.edu.cn; weishe@seu.edu.cn; zmsun@seu.edu.cn

Part 1. Figures

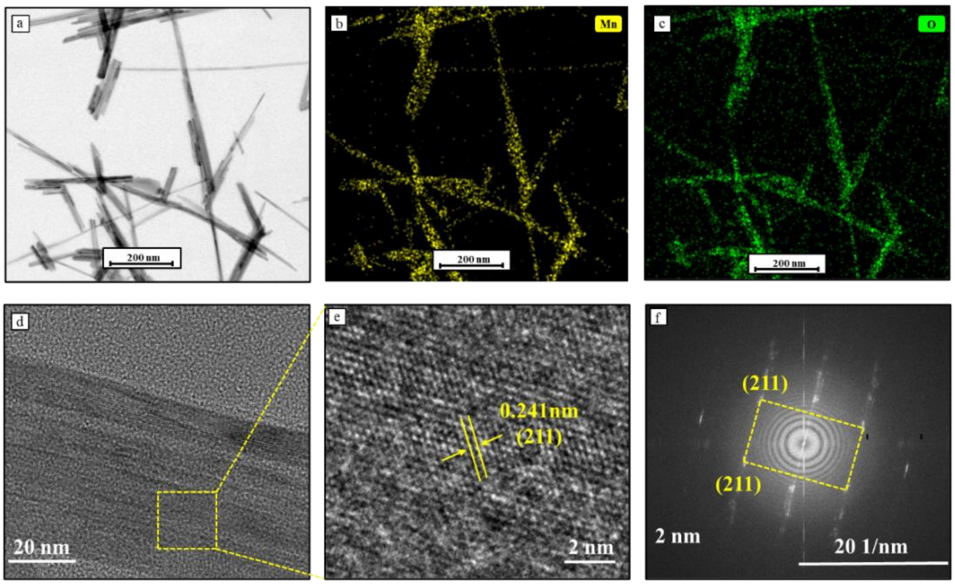

Figure S1. a) TEM image, b-c) elemental mapping images, d-e) HR-TEM images, and f) SAED pattern of MnO_2.

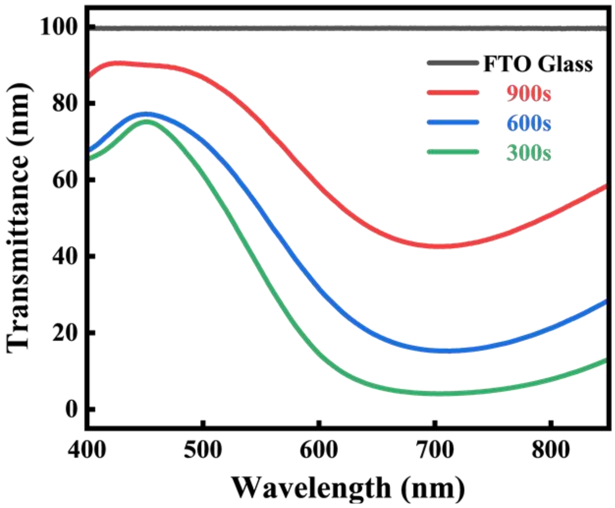

Figure S2. UV-vis spectra of PB electrodes with electrochemical deposition times of 300s, 600s, and 900s at -15μA cm^{-2} electrodeposition current density, the transmittance of the FTO glass or ITO/PET was used as the baseline during the measurement.

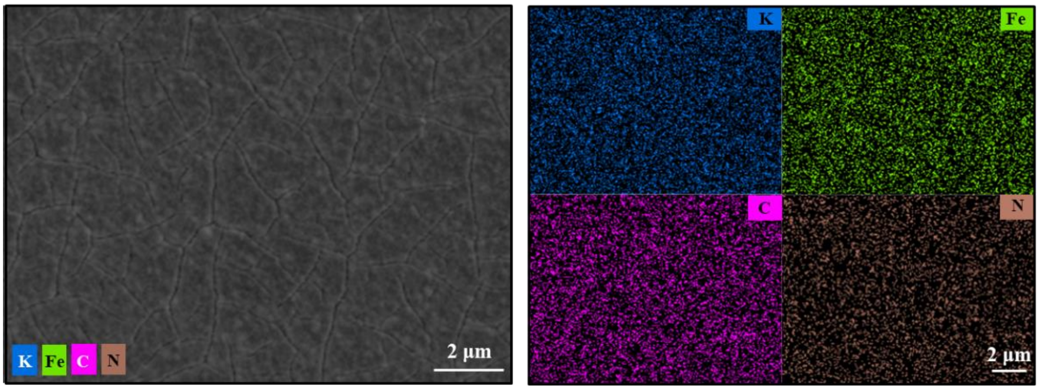

Figure S3. EDS mapping of the PB electrode and the compositional distributions of K, Fe, C and N elements.

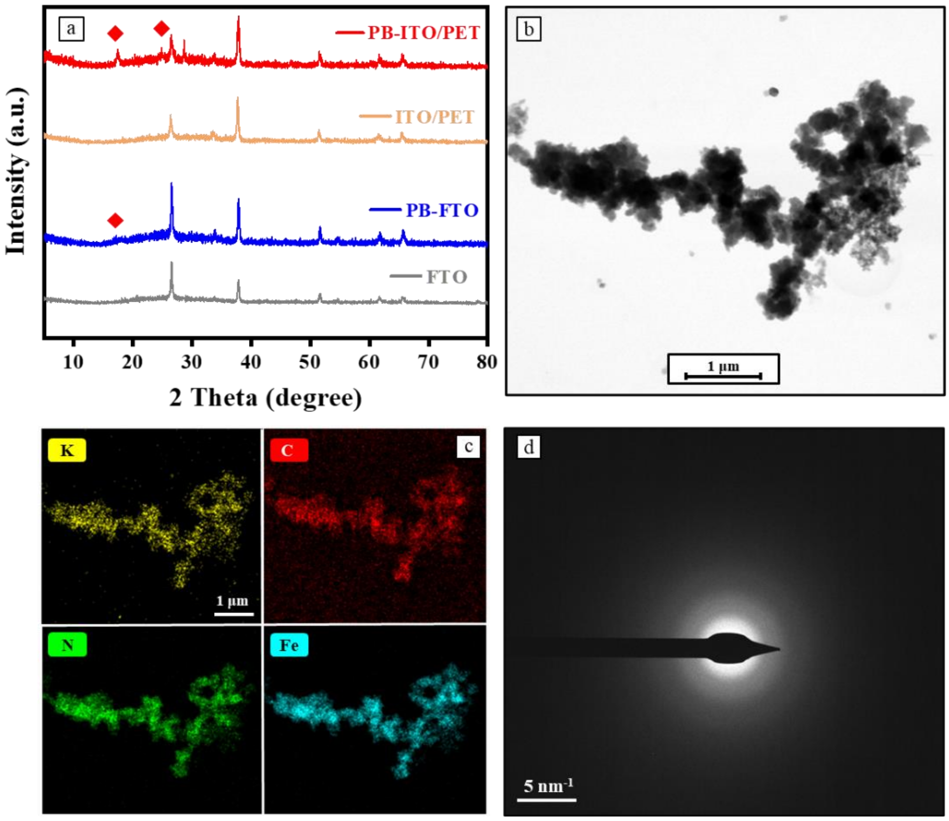

Figure S4. a) XRD patterns of FTO, PB-FTO, ITO/PET, and PB-ITO/PET, b) TEM image, c) elemental mapping images, d-e) HR-TEM images and f) SAED pattern of PB powder. The PB samples are scraped from the PB-FTO glass.

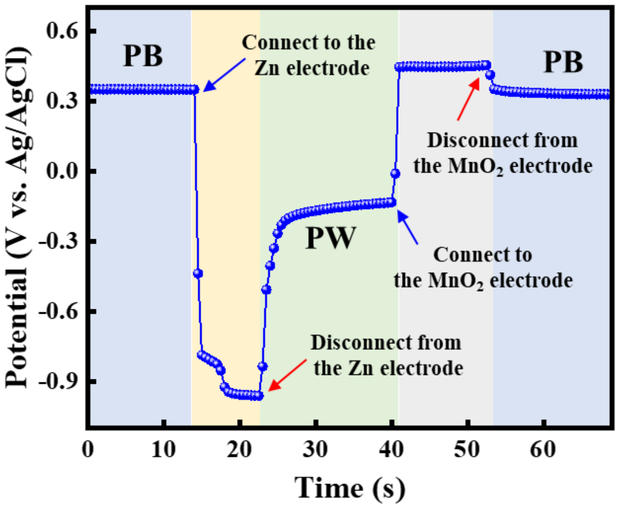

Figure S5. In situ potential measurement of the PB electrode when it connects to Zn electrode and ECP electrode in turn. When the PB electrode connects to the Zn electrode, the potential rapidly decreases as electrons flow from the Zn electrode to the PB electrode, resulting in the reduction of Fe^{3+} to Fe^{2+} and the transformation of the blue PB electrode into the colorless PW electrode. Upon connecting the PW electrode to the MnO_2 electrode, electrons flow from the PW electrode to the MnO_2 electrode, causing an increase in the potential. Following disconnection from the MnO_2 electrode, the potential returns to its initial state.

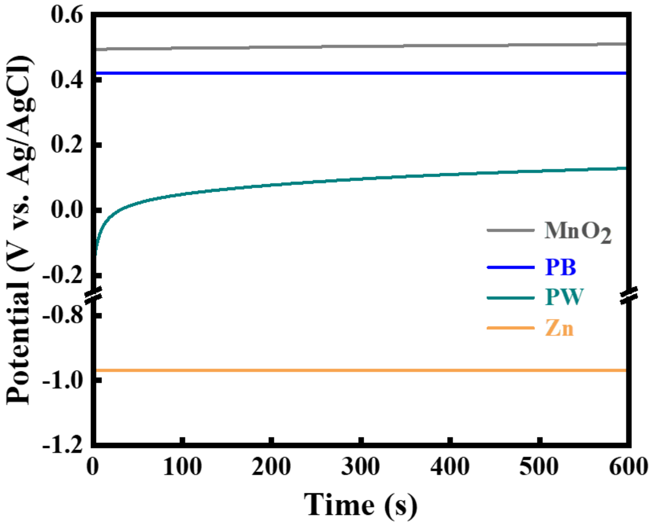

Figure S6. Potentials of Zn, PB, PW and MnO_2 electrodes in the electrolyte containing 2M $ZnSO_4$, 0.2M $MnSO_4$ and 0.1M K_2SO_4.

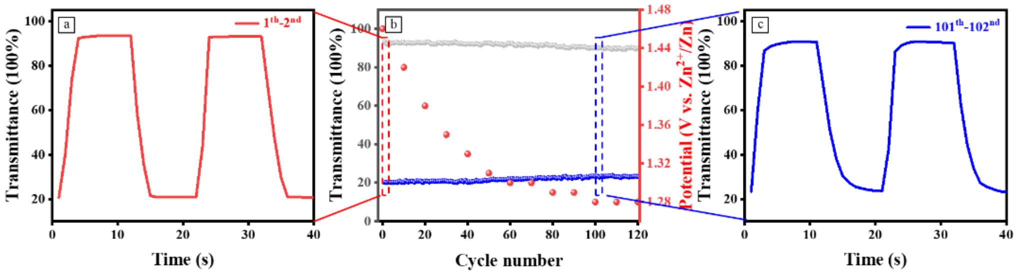

Figure S7. a) The switching speed of the 1th and 2nd electrochromic cycle; b) The synchronized transmittance profile and the overall cell potential; c) The switching speed of the 101th and 102nd electrochromic cycle, the switching times are set to 10 s.

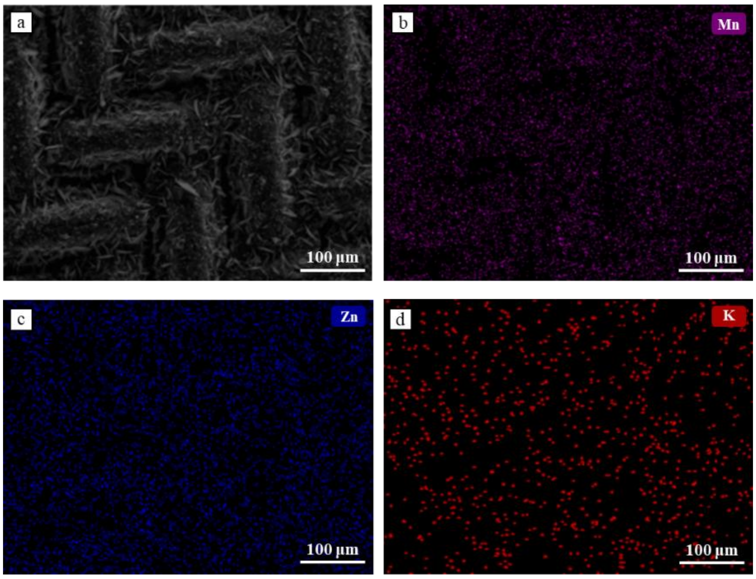

Figure S8. a) The SEM image and the corresponding EDS mapping of the post-electrochromic (discharged) MnO_2 electrode, revealing a significant presence of Zn and K elements, and after charging, most Zn elements and all K elements disappeared.

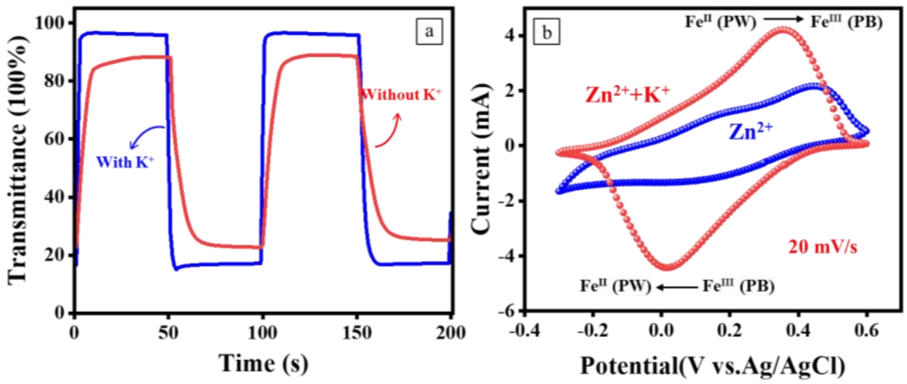

Figure S9. a) In situ transmittance measurement of the Zn||PB||MnO_2 electrochromic system in the electrolyte with/without K^+; b) CV curves of the PB film in the electrolyte with and without K^+.

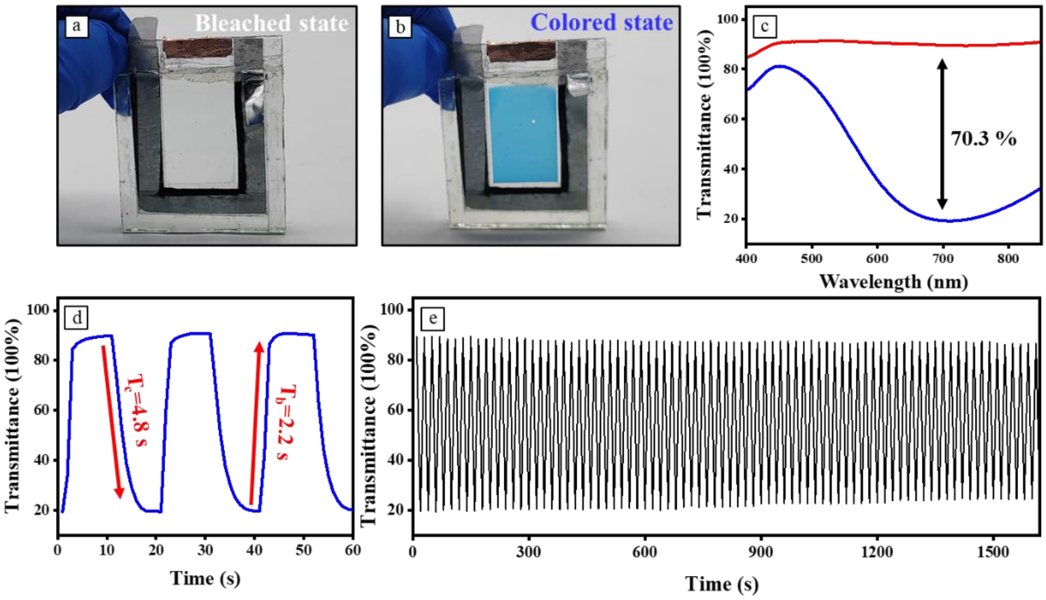

Figure S10. Electrochromic performance of the real air-working Zn||PB||MnO_2 electrochromic system. a) and b) Optical photos of bleached state and colored state of PB electrode; c) UV-vis transparence spectra of PB and PW; d) In situ transmittance measurement at 700nm of PB connected with Zn electrode and MnO_2 electrode for 10 s, respectively; e) In situ transmittance measurement of Zn||PB||MnO_2 system under repeated bleaching and coloring cycles, the switching time is set to 10 s.

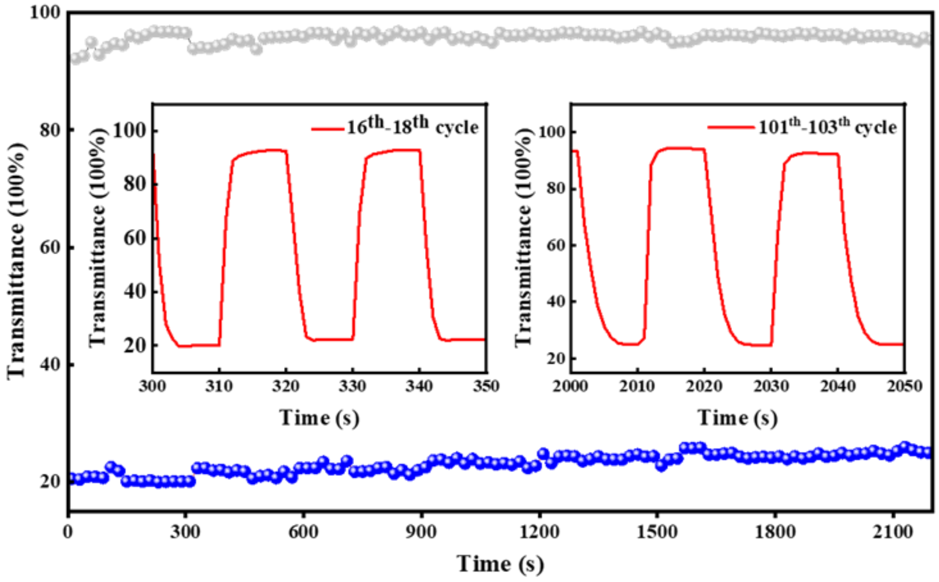

Figure S11. In situ transmittance measurement of Zn||PB||MnO_2 system under repeated bleaching and coloring cycles after repeating charging and electrochromic-discharging for 50 times, the switching time is set to 10s.

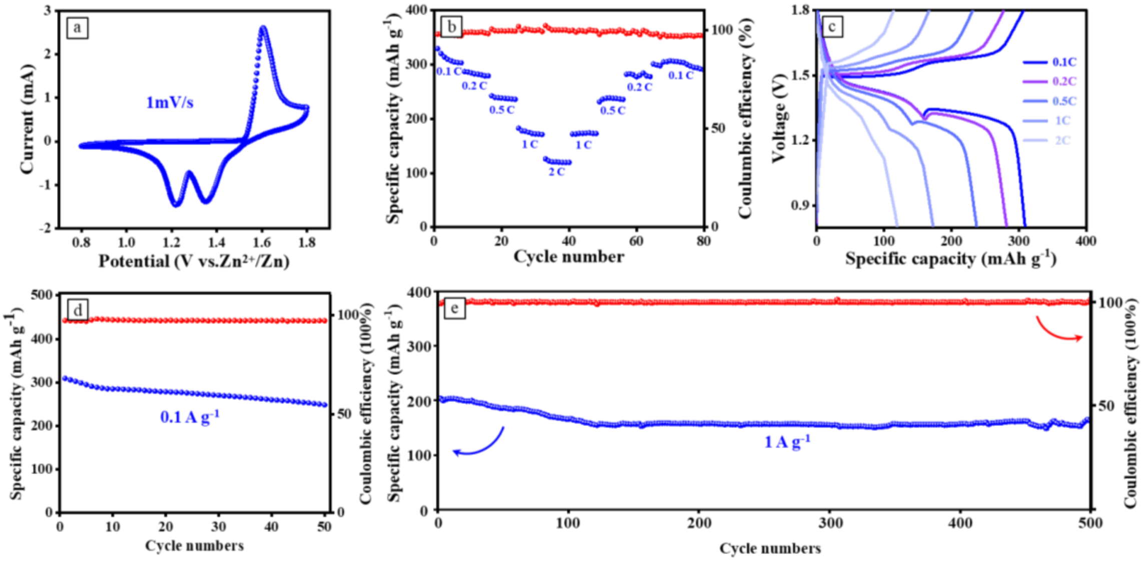

Figure S12. a) Cyclic voltammetry curve of MnO_2 cathode at a scan rate of 1 mV s^{-1}; b) The rate capability and c) Charge-discharge profiles of the Zn/MnO_2 battery; Cyclic performance of Zn/MnO_2 battery at d) 0.1 A g^{-1} and e) 1 A g^{-1}.

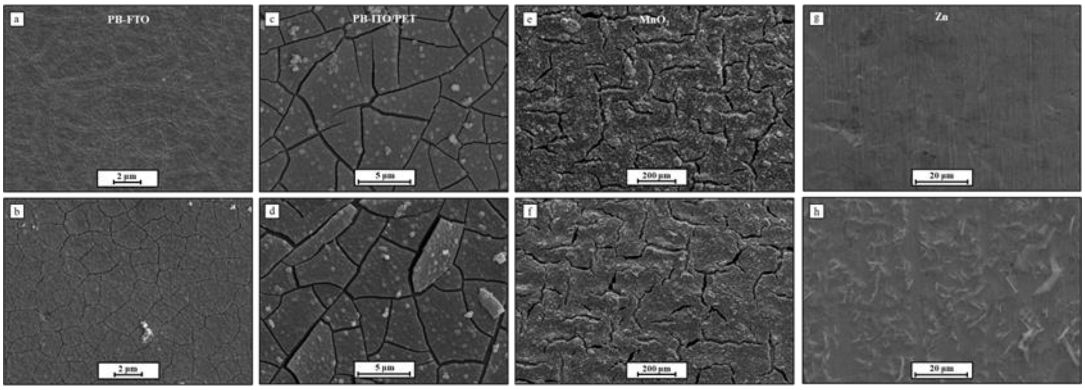

Figure S13. The initial and after-cycling SEM images of a-b) PB/FTO, c-d) PB-ITO/PET; e-f) Zn, and g-h) MnO_2 electrodes.

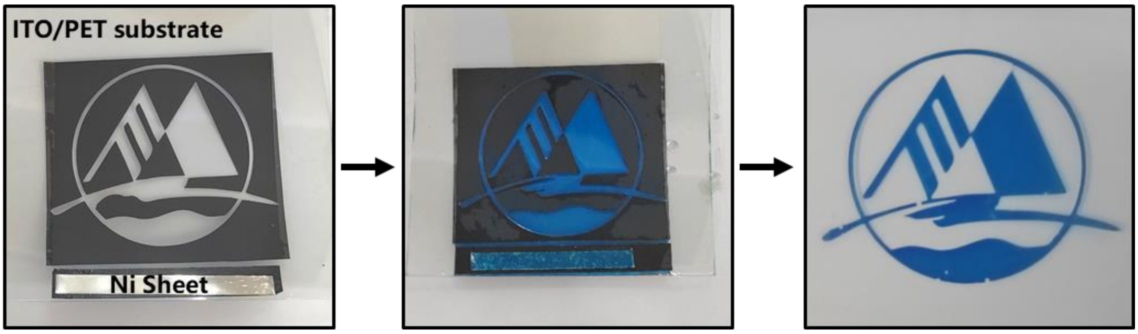

Figure S14. Preparation of the electrochromic label utilizing galvanic-driven deposition method. Briefly, A piece of metallic nickel was tightly attached to the conductive side of the ITO/PET substrate through a conductive double-sided tape, and then immersed into a

homogeneous solution containing 10 mM $K_3[Fe(CN)_6]$, 10 mM $FeCl_3$ and 50 mM KCl for about 120 s. Then the PB/ITO electrode was flushed with deionized water and dried at 70 °C.

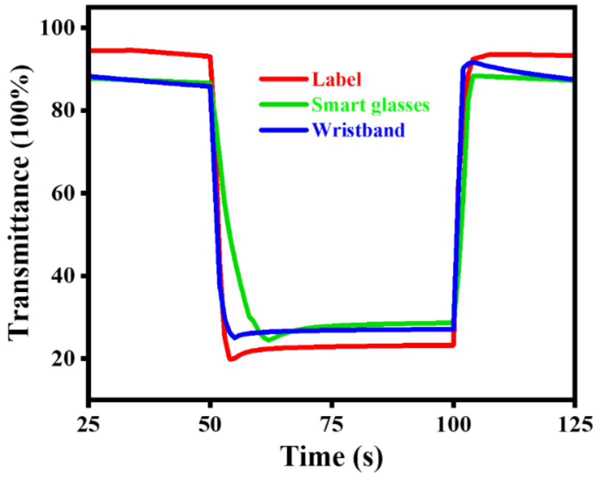

Figure S15. In situ transmittance measurement of the electrochromic label, glasses, and wristband.

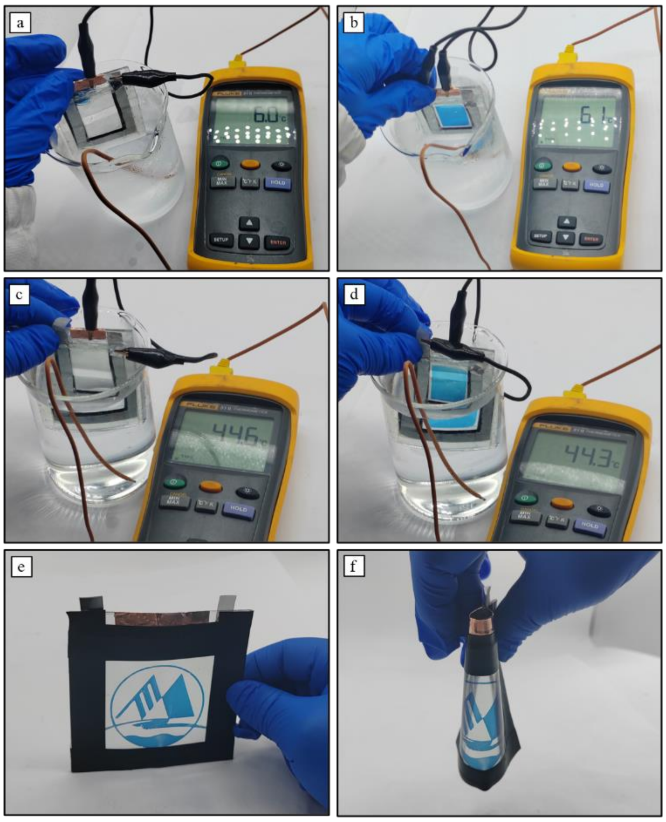

Figure S16. The electrochromic label operates at ice/hot water, a-b) Bleached and colored states at ~6°C; c-d) Bleached and colored states at ~44°C; The electrochromic label in e) flat and f) folded states.

Part 2. Tables

Table S1. Comparison of the optical contrast and switching speed of our Zn||PB||MnO_2 system with recently reported electrochromic systems.

Electrochromic System	Bleaching Speed (s)	Coloring Speed (s)	Optical contrast	Self-powered (Y/Half/N)	Reference
WO_{3-x}	3.7	4.5	76	N	[1]
P-WO_3	6.4	1.7	40	N	[2]
WO_3/Ag HMG film	14.7	6.8	53	N	[3]
$W_{17}O_{47}$ @PEDOT:PSS	3.5	5.5	79.7	N	[4]
$Nb_{18}W_{16}O_{93}$	4	4.7	53.1	N	[5]
$W_{18}O_{49}$ nanowires	4.1	4.5	70	N	[6]
TiO_2/MXene	1.71	0.36	41.09	N	[7]
$Li_4Ti_5O_{12}$	4.35	7.65	74.73	N	[8]
ZnO@Ni/Co-LDH	4.7	5.8	63	N	[9]
Bi/Cu reversible electrodeposition	2.34	11.12	63	N	[10]
CuSn reversible electrodeposition	18.4	9.8	79.33	N	[11]
PANI	16.9	5.9	~ 60	N	[12]
HOF	4.4	5.6	66	N	[13]
Ni-BTA	1.8	5	65.9	N	[14]
Viologen	1.2	2.8	68.6	N	[15]
$LiMn_2O_4$||WO_3	12.5	10.2	30	N	[16]
α-MnO_2||m-WO_3	10.5	11.4	65.7	N	[17]
W/WO_3||WO_3	2.5	2.4	53.6	N	[18]
Zn||PB	4.7	7.5	70	Half	[19]
Al||PB	4.1	4.6	52.2	Half	[20]
Zn||WO_3	10.3	5.7	77	Half	[21]
Zn mesh||PB	2.5	3.6	67.2	Half	[22]
Zn||WO_{3-x}	2.5	3.5	55	Half	[23]
Zn||PB||ECP	1	2.2	68	Y	[24]
WO_3||Zn||PB	3.8	4	72.6	Y	[25]
Mg||PB||MnO_2	4.3	4.8	> 80	Y	[26]
**Zn||PB||MnO_2**	**2.0**	**3.2**	**80.6**	**Y**	**This work**

Note: "Half" means that these electrochromic systems can only achieve a unidirectional coloring or bleaching transition, while still need to be connected to an external power supply to revert to their initial states.

Part 3. Movies

Movie S1 shows the self-bleaching/coloring processes of the Zn||PB||MnO_2 electrochromic system. When connecting the PB electrode to Zn in the Zn||PB||MnO_2 system, electrons flow from the Zn electrode to the PB electrode, reducing Fe^{3+} to Fe^{2+} and leading to the transformation of the blue PB electrode to the colorless PW electrode. After the PW electrode is switched to connect with MnO_2 electrode, electrons flow from PW electrode to MnO_2 electrode and lead to blue PB. Both the bleaching and coloring processes are driven by the potential difference between Zn/PB electrodes and PW/MnO_2 electrodes. Movie S2-S4 demonstrate the self-powered bleaching and coloring switching of the wearable electrochromic glasses, labels, and wristband, respectively. Movie S5 demonstrates the electrochromic performance of the real air-working Zn||PB||MnO_2 electrochromic system. Movie S6 shows the electrochromic label in flat and folded states.

Reference:

[1] L. Zhang, D. Chao, P. Yang, L. Weber, J. Li, T. Kraus, H. J. Fan, Flexible Pseudocapacitive Electrochromics via Inkjet Printing of Additive-Free Tungsten Oxide Nanocrystal Ink. Adv. Energy Mater. 10, 2000142 (2020).

[2] W. C. Poh, A. L. Eh, W. Wu, X. Guo, P. S. Lee, Rapidly Photocurable Solid-State Poly(ionic liquid) Ionogels For Thermally Robust and Flexible Electrochromic Devices. Adv. Mater. 34, 2206952 (2022).

[3] T. Li, S. Li, X. Li, X. Zhang, J. Zhao, Y. Shi, Y. Wang, R. Yu, X. Li, Q. Xu, W. Guo, A leaf vein-like hierarchical silver grids transparent electrode towards high-performance flexible electrochromic smart windows. Sci. Bull. 65, 225-232 (2020).

[4] Q. Zhao, J. Wang, X. Ai, Y. Duan, Z. Pan, S. Xie, J. Wang, Y. Gao, Three-dimensional knotting of $W_{17}O_{47}$@PEDOT : PSS nanowires enables high-performance flexible cathode for dual-functional electrochromic and electrochemical device. InfoMat. 4, e12298 (2022).

[5] C. Wu, Z. Shao, W. Zhai, X. Zhang, C. Zhang, C. Zhu, Y. Yu, W. Liu, Niobium Tungsten Oxides for Electrochromic Devices with Long-Term Stability. ACS Nano.16, 2621-2628 (2022).

[6] S. Sheng, J. Wang, B. Zhao, Z. He, X. Feng, Q.-G. Shang, C. Chen, G. Pei, J. Zhou, J. Liu, S. Yu, Nanowire-based smart windows combining electro- and thermochromics for dynamic regulation of solar radiation. Nat. Commun. 14, 3231 (2023),

[7] R. Li, X. Ma, J. Li, J. Cao, H. Gao, T. Li, X. Zhang, L. Wang, Q. Zhang, G. Wang, C. Hou, Y. Li, T. Palacios, Y. Lin, H. Wang, X. Ling, Flexible and high-performance electrochromic devices enabled by self-assembled 2D TiO_2/MXene heterostructures. Nat. Commun. 12, 1587 (2021).

[8] Z.-S. Wu, Z.-D. Lian, Y. Su, J. Li, J. Xu, S. Chen, Z. Tang, S. Wang, K. W. Ng, Extraordinarily Stable Aqueous Electrochromic Battery Based on $Li_4Ti_5O_{12}$ and Hybrid Al^{3+}/Zn^{2+} Electrolyte. ACS Nano. 16, 13199-13210 (2022).

[9] X. Liu, J. Wang, D. Tang, Z. Tong, H. Ji, H. Qu, , A forest geotexture-inspired ZnO@Ni/Co layered double hydroxide-based device with superior electrochromic and energy storage performance. J. Mater. Chem. A. 10, 12643-12655 (2022).

[10] C. Su, Z. Zhao, D. He, H. Song, C. Zhao, W. Mai, Five-state flexible dynamic windows. Nano Energy. 111, 108396 (2023).

[11] A. L. Eh, J. Chen, S. H. Yu, G. Thangavel, X. Zhou, G. Cai, S. Li, D. H. C. Chua, P. S. Lee, A Quasi-Solid-State Tristate Reversible Electrochemical Mirror Device with Enhanced Stability. Adv. Sci. 7, 1903198 (2020).

[12] Q. Wang, S. Cao, Q. Meng, K. Wang, T. Yang, J. Zhao, B. S. Zou, Robust and stable dual-band electrochromic smart window with multicolor tunability. Mater. Horiz. 10, 960-966 (2023).

[13] J. Feng, Y. Luo, X. Wang, G. Cai, R. Cao, A Large-Area Patterned Hydrogen-Bonded Organic Framework Electrochromic Film and Device. Small. 2304691 (2023).

[14] G. Cai, P. Cui, W. Shi, S. Morris, S. N. Lou, J. Chen, J. Ciou, V. K. Paidi, K. Lee, S. Li, P. S. Lee, One-Dimensional π–d Conjugated Coordination Polymer for Electrochromic Energy Storage Device with Exceptionally High Performance. Adv. Sci. 7, 1903109 (2020).

[15] Z. Wang, X. Jia, P. Zhang, Y. Liu, H. Qi, P. Zhang, U. Kaiser, S. Reineke, R. Dong, X. Feng, Viologen-Immobilized 2D Polymer Film Enabling Highly Efficient Electrochromic Device for Solar-Powered Smart Window. Adv. Mater. 34, 2106073 (2022).

[16] L. Liu, X. Diao, Z. He, Y. Yi, T. Wang, M. Wang, J. Huang, X. He, X. Zhong, K. Du, High-performance all-

inorganic portable electrochromic Li-ion hybrid supercapacitors toward safe and smart energy storage. Energy Stor. Mater. 33, 258-267 (2020).

[17] C. Deng, K. Zhang, L. Liu, Z. He, J. Huang, T. Wang, Y. Liu, X. He, K. Du, Y. Yi, High-performance all-solid-state electrochromic asymmetric Zn-ion supercapacitors for visualization of energy storage devices. J. Mater. Chem. A. 10, 17326-17337 (2022).

[18] Y. Li, P. Sun, J. Chen, X. Zha, X. Tang, Z. Chen, Y. Zhang, S. Cong, F. Geng, Z. Zhao, Colorful Electrochromic Displays with High Visual Quality Based on Porous Metamaterials. Adv. Mater. 35, 2300116 (2023).

[19] Y. Ding, H. Sun, Z. Li, C. Jia, X. Ding, C. Li, J. Wang, Z. Li, Galvanic-driven deposition of large-area Prussian blue films for flexible battery-type electrochromic devices. J. Mater. Chem. A. 11, 2868-2875 (2023).

[20] J. Wang, L. Zhang, L. Yu, Z. Jiao, H. Xie, X. W. Lou, X. Wei Sun, A bi-functional device for self-powered electrochromic window and self-rechargeable transparent battery applications. Nat. Commun. 5, 4921 (2014).

[21] H. Li, C. J. Firby, A. Y. Elezzabi, Rechargeable aqueous Hybrid Zn^{2+}/Al^{3+} Electrochromic batteries. Joule. 3, 2268-2278 (2019).

[22] H. Li, W. Zhang, A. Y. Elezzabi, Transparent Zinc-Mesh Electrodes for Solar-Charging Electrochromic Windows. Adv. Mater. 32, 2003574 (2020).

[23] J. Li, P. Yang, X. Li, C. Jiang, J. Yun, W. Yan, K. Liu, H. J. Fan, S. W. Lee, Ultrathin Smart Energy-Storage Devices for Skin-Interfaced Wearable Electronics. ACS Energy Lett. 8, 1-8 (2022).

[24] Y. Luo, H. Jin, Y. Lu, Z. Zhu, S. Dai, L. Huang, X. Zhuang, K. Liu, L. Huang, Potential Gradient-Driven Fast-Switching electrochromic device. ACS Energy Lett. 7, 1880-1887 (2022).

[25] W. Zhang, H. Li, A. Y. Elezzabi, A Dual-Mode Electrochromic Platform Integrating Zinc Anode-Based and Rocking-Chair Electrochromic Devices. Adv. Funct. Mater. 33, 2300155 (2023).

[26] Q. Ma, J. Chen, H. Zhang, Y. Su, Y. Jiang, S. Dong, Dual-Function Self-Powered Electrochromic Batteries with Energy Storage and Display Enabled by Potential Difference. ACS Energy Lett. 8, 306-313 (2022).
